# The case of a 49‐year‐old man with involuntary toe movements

**DOI:** 10.1002/acn3.51778

**Published:** 2023-06-15

**Authors:** Gabriela F. Pucci, Aaron L. Berkowitz

**Affiliations:** ^1^ Department of Neurology University of Pittsburgh medical center Pittsburgh; ^2^ Department of Neurology University of California San Francisco San Francisco California

**Keywords:** movement disorders, painful legs and moving toes, polyneuropathy, work ability

## Abstract

A 49‐year‐old man presented with 3 years of leg pain and involuntary toe movements. He described the pain as mild burning, radiating from the left foot upward to the leg. On examination, there were involuntary continuous flexion‐extension movements of his left toes (video). Strength, sensation, and reflexes were normal. Lumbosacral MRI demonstrated diffuse degenerative disc disease with multi‐level mild‐to‐moderate foraminal stenosis. Nerve conduction studies were normal. EMG showed neurogenic potentials and active denervation changes in the left anterior tibial and soleus muscles consistent with radiculopathy. The diagnosis of painful legs and moving toes is discussed.

## Diagnosis

Painful legs and moving toes (PLMT).

## Take‐Home Points

- PLMT is characterized by lower extremity pain with continuous involuntary movements (flexion‐extension, adduction‐abduction, or “continual wriggling and writhing.”)^1^, ^2^
- PLMT is often caused by radiculopathy or neuropathy^3^
- The pain of PLMT can be treated with medications for neuropathic pain; the toe movements with clonazepam, pramipexole, ropinirole, or botulinum toxin.^2^

See Video S1 attached to the submission page, print screen below.

## Supporting information


**Video S1.** Continuous, involuntary flexion‐extension movements of the left toes.
